# Response of the characteristics of organic carbon mineralization of soft rock and soil composed of sand to soil depth

**DOI:** 10.7717/peerj.11572

**Published:** 2021-06-04

**Authors:** Wanying Li, Zhen Guo, Juan Li, Jichang Han

**Affiliations:** 1 College of Land Engineering, Chang’an University, Xi’an, Shaanxi, China; 2 Shaanxi Provincial Land Engineering Construction Group Co., Ltd., Xi’an, Shaanxi, China; 3 Institute of Land Engineering and Technology, Shaanxi Provincial Land Engineering Construction Group Co., Ltd., Xi’an, Shaanxi, China

**Keywords:** Mineralization, Soil depth, Activated organic carbon, Compound soil, Mu Us sandy land

## Abstract

The addition of soft rock to aeolian sandy soil can improve the level of fertility and ability of the soil to sequester carbon, which is of substantial significance to improve the ecological environment of the Mu Us sandy land and supplement newly added cultivated land. S oft rock and sand were combined using the ratio (v/v) of 0:1 (CK), 1:5 (S1), 1:2 (S2), and 1:1 (S3). The process of mineralization of organic carbon at different depths (0–10 cm, 10–20 cm, and 20–30 cm) in the combined soil was studied by 58 days of incubation indoors at a constant temperature. The content of soil nutrient s increased significantly under the S2 and S3 treatments and was higher in the 0–10 cm soil depth. The mineralization of rate of soil organic carbon (SOC) of different combination ratios can be divided into three time periods: the stress mineralization stage (1–7 d), the rapid mineralization stage (7–9 d) and the slow mineralization stage (9–58 d). At the end of incubation, the rates of mineralization of SOC and accumulated mineralization amount (C_t_) were relatively large in the 0–10 cm soil depth, followed by the 10–20 cm and 20–30 cm soil layers , indicating that the stability of SOC in the surface layer was poor, which is not conducive to the storage of carbon. The content of potentially mineralizable organic carbon (C_0_) in the soil was consistent with the trend of change of C_t_. Compared with the CK treatment, the cumulative organic carbon mineralization rate (C_r_) of the S2 and S3 treatment s decreased by 7.77% and 6.05%, respectively; and the C_0_/SOC decreased by 22.84% and 15.55%, respectively. Moreover, the C_r_ and C_0_/SOC values in the 10–20 cm soil depth were small, which indirectly promoted the storage of organic carbon. With the process of SOC mineralization, the contents of soil microbial biomass carbon (SMBC) and dissolved organic carbon (DOC) tended to decrease compared with the initial contents, with larger amplitudes in the 20–30 cm and 10–20 cm soil depth s, respectively. SOC, total nitrogen, available potassium, SMBC and DOC were all closely related to the process of mineralization of organic carbon. Therefore, the accumulation of soil carbon could be enhanced when the proportion of soft rock and sand composite soil was between 1:2 and 1:1, and the 10–20 cm soil depth was relatively stable. These results provide a theoretical basis for the improvement of desertified land.

## Introduction

Soil organic carbon (SOC), an important component of soil, plays a critical role in soil fertility, ecological system balance, and sustainable agricultural development, although it only comprises a small part of the total soil quality (Bishwakarma et al., 2014). SOC refers to the total amount of organic matter in the soil that contains carbon, which is primarily composed of plant and animal residues, soil humus and soil microbial carbon (Martens, Reedy & Lewis, 2004; Gomez, Delgado & Gonzalez, 2021). The SOC pool is twice that of the atmospheric carbon pool and three times the total carbon pool of the earth’s vegetation, and approximately 80% of the total carbon involved in the carbon cycle of the earth’s land area exists in the soil in the form of SOC (Körschens, 2010; Ganjegunte et al., 2005). Small changes in the soil carbon pool can lead to significant changes in the concentration of atmospheric CO_2_, indicating that the fixation and mineralization of SOC play an important regulatory role in the concentration of global atmospheric CO_2_ (Kuzyakov, Friedel & Stahr, 2000; Guo et al., 2021). In this context, it is highly important to understand the dynamic changes in the global terrestrial ecosystem carbon pool to reduce the greenhouse effect and improve the quality of environment (Moore et al., 2020).

The mineralization of SOC is an important biochemical process in the soil, which not only reflects the stability and turnover rate of organic carbon but also directly relates to the release and supply of soil nutrients (Marinari et al., 2010). The process of SOC mineralization is affected by many factors, such as the chemical composition and existence of soil organic matter, soil microbial population composition and activity, soil physical and chemical properties, soil type, and soil depth thickness (Saggar, 2001; Huang et al., 2002). Among them, the thickness of soil depth is an important factor that affects the mineralization of organic carbon, and the rate of mineralization of organic carbon of the soil below the surface has a significant impact on the atmospheric circulation effect (Fontaine et al., 2007). Jobbagy & Jackson (2000) reported that in the global terrestrial ecosystem, the carbon content of soil at a depth of 20–300 cm is three times higher than that of the surface soil. In the context of global climate change, plant roots grow to deeper soils, and the production of root exudates and the input of animal and plant residues will change the carbon input at deeper soil depths, thereby triggering the mineralization and decomposition of carbon (Lal et al., 2007). Karhu et al. (2016) studied the ability of organic carbon to stimulate mineralization at different depths of boreal forest soils and showed that the mineralization of organic carbon in the lower soil is more sensitive to that of external carbon. However, Paterson & Sim (2013) found that the mineralization of organic carbon in the upper and lower layers of the soil responds to outside factors in the same way. When there is no stimulation, some recent studies have shown that deep soil organic carbon is more stable than surface organic carbon (Schimel et al., 2011). In farmland and woodland ecosystems, studies have shown that soil depth, sampling method, and cultivation time have significant effects on the mineralization of organic carbon, while temperature has no obvious effect (Fang et al., 2005). Thus, without the addition of external carbon sources, as the roots of crops gradually grow to the lower layer, the intensity of mineralization of the soil itself on the vertical scale merits further clarification.

In the semiarid Mu Us sandy land in northern China, the development of desertification has led to the fragmentation of habitats and patchy distribution of vegetation, as well as the depletion of nutrients and a high degree of spatial heterogeneity in their distribution (Liu et al., 2020). By studying the soft rock and aeolian sandy soil (sand) in the Mu Us sandy land, Han, Liu & Luo (2012) obtained a formula for the combination of soft rock and sand suitable for different crop growth needs. The use of soft rock to improve sandy soil not only enhances the air permeability and water retention of sand but also increases crop yields and promotes the development of sandy soil to loamy soil (Wang et al., 2016; Guo, Zhang & Wang, 2020; Li et al., 2019). Guo, Han & Li (2020) showed that non-dominant bacteria in the 0–30 cm soil depth play an important role in the fixation of organic carbon, but the influence of soil thickness on the mineralization of organic carbon in the compound soil has not been studied. The soft rock and sand composite soil is a mixed soil depth (0–30 cm) that is utilized in engineering measures, such as excavation and filling, crushing soft rock, and topsoil stripping. Since microorganisms in the mixed soil depth are affected by the external environment and root growth, there must be a gradient or differences (Sun & Han, 2018). However, in the 0–30 cm mixed soil depth, under the action of microorganisms, the degree of mineralization of organic carbon of the composite soil every 10 cm interval remains to be elucidated. Therefore, this study aimed to understand the potential of soft rock and sandy composite soil to undergo mineralization at different soil depths using laboratory soil incubation experiments and to understand the biological feedback mechanism of desertification from the perspective of soil carbon mineralization that affects nutrient cycling. Therefore, the purpose of this study was to (1) study the difference in gradients of contents of soil nutrients between mixed soil depths; (2) explore the intensity of mineralization of organic carbon in soft rock and sand composite soil at different soil depths; and (3) reveal the relationship between mineralization of organic carbon and nutrients at different soil depth. We hypothesized the following outcomes: (1) the compound soil at a depth of 0–10 cm had a higher nutrient content; (2) the compound soil was more stable and had could sequester carbon more effectively at a depth of 10–20 cm, and (3) the turnover time of carbon pool was longer, and the content of nutrients was higher when the proportion of addition of soft rock to the sandy soil was between 33.33–50%.

## Materials & Methods

### Study site

The experimental plot was established in a pilot test base of the Shaanxi Provincial Land Engineering Technology Research Institute (Fuping county, China) (109°11′E, 34°42′N). The area is in the transition zone between the Guanzhong Plain and the northern Shaanxi Plateau and belongs to the gully region of the Weibei Loess Plateau. The terrain is high in the north and low in the south. It slopes from northwest to southeast. The elevation in the territory is 375.8–1, 420.7 m. The area belongs to the continental monsoon warm zone and has a semiarid climate. The annual total radiation is 5,187.4 MJ m^−2^; the annual average sunshine hours is approximately 2,389.6 h; the annual average temperature is 13.1 °C, and the annual average precipitation is 527.2 mm. The interannual precipitation varies substantially, and its coefficient of variation (CV) reaches 21.1%.

### Experimental design

**Figure 1:**
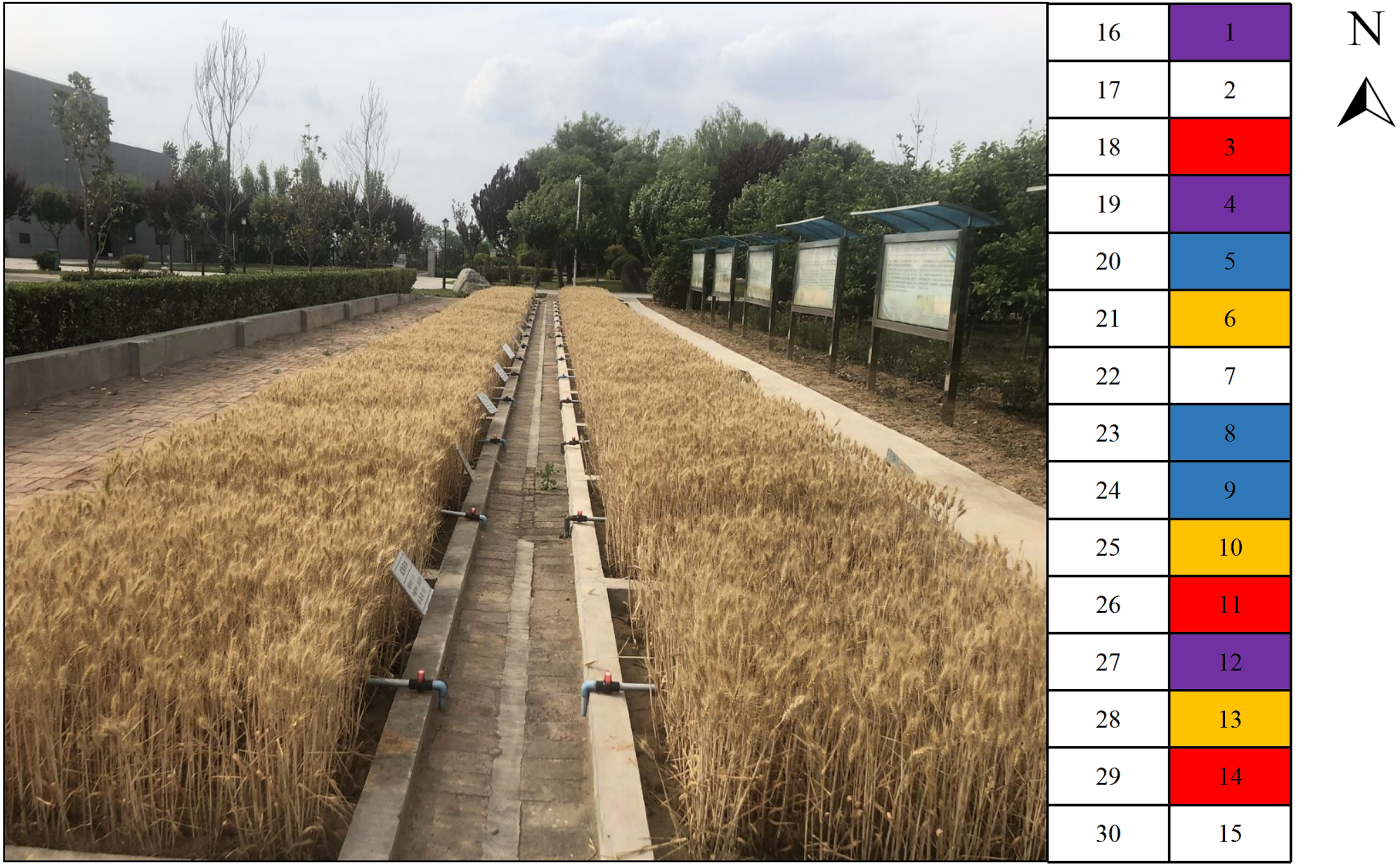
The test field of soft rock and sand compound soil. Test field 1 to 15 were set in 2009 and 16 to 30 were set in 2016. The field 2, 7, 15, 17, 22 and 30 represent that the volume ratio of loess to sand is 1:2, and apply organic fertilizer. The field 6, 10, 13, 21, 25 and 28 represent that the volume ratio of soft rock to sand is 1:1, the field 5, 8, 9, 20, 23 and 24 represent that the volume ratio of soft rock to sand is 1:2, the field 1, 4, 12, 16, 19 and 27 represent that the volume ratio of soft rock to sand is 1:5, the field 3, 11, 14, 18, 26 and 29 represent that the volume ratio of soft rock to sand is 0:1. The color-marked test field are selected for this trial. The red area represent CK treatment, the purple area represent S1 treatment, the blue area represent S2 treatment, and the yellow area represent S3 treatment.

The test field was utilized to simulate the conditions of soft rock and sand mixed layer in the Mu Us sandy land. The design depth of the experimental field was 1 m, and it was filled with a mixture of soft rock and sand at a depth of 0–30 cm and filled with aeolian sandy soil at a depth of 30–70 cm. The test was conducted in 2009, and four treatments of soft rock and sand in a volume ratio of 0:1 (CK), 1:5 (S1), 1:2 (S2), and 1:1 (S3) were selected. Each treatment was repeated three times for a total of 12 trial plots (Fig. 1). The area of each plot was 2 m × 2 m. Based on the site conditions of the test plot, considering the uniformity of factors, such as illumination and microtopography, the test plot was arranged from south to north in a “one” shape. The experimental field was artificially sown with was maize (Jincheng 508) and wheat (Xiaoyan 22) with a rotation of two crops a year. The types of fertilizers tested in the experimental field were urea, including N 46.4%, diammonium phosphate; including N 16%, containing P_2_O; 44%, potassium sulfate, including K_2_O 52%, and the amount of fertilizer applied was 255 kg hm^−2^ (N), 180 kg hm^−2^ (P_2_O_5_) and 90 kg hm^−2^ (K_2_O). The mode of fertilization of each treatment was the same, and all three fertilizers were applied.

### Collection of soil samples

After the wheat was harvested in May 2020, soil samples at a depth of 0–30 cm (0–10 cm, 10–20 cm and 20–30 cm) were collected from each plot. Soil samples were collected from five different points and mixed to make a composite sample for each plot. The animal and plant residues were removed from the collected soil samples, sieved through a 1-mm sieve, and then divided into two portions. One was placed at 4 °C for the mineralization incubation test, and one was naturally air-dried to determine the basic physical and chemical properties.

### Method of determination

The soil organic carbon (SOC) was determined using the potassium dichromate-concentrated sulfuric acid external heating method as previously described (Nelson & Sommers, 1996). The total nitrogen (TN) was determined using Kjeldahl digestion; the available phosphorus (AP) was determined using molybdate blue colorimetry, and the concentrations of available potassium (AK) were measured using atomic absorption spectrometry (Qiu et al., 2014). The soil particle composition was determined using a Mastersizer 3000 laser diffraction particle size analyzer (Malvern Instruments, Worcestershire, UK) (Sun & Han, 2018).

The content of dissolved organic carbon (DOC) content was determined by extraction with deionized water as previously described (Ghani, Dexter & Perrott, 2003). The specific steps were as follows: the weight of fresh soil equivalent to that of 10 g of dry soil was placed in a 50 mL centrifuge tube, and 30 mL of deionized water was added. The mixture was shaken and extracted for 30 min at room temperature and centrifuged using a high-speed centrifuge (Allegra 64R; Beckman Coulter, New York City, NY, USA). After centrifugation, the supernatant was passed through a 0.45-µm filter membrane, and the filtrate was measured with a Multi N/C 3100 total organic carbon/total nitrogen analyzer (AnalytikJena, Jena, Germany). The soil microbial biomass carbon (SMBC) was determined using the chloroform fumigation-potassium sulfate extraction method as previously described (Brookes et al., 1985).

The organic carbon mineralization test utilized the lye absorption method (Ribeiro et al., 2010), which entailed weighing 30 g of the soil stored at 4 °C into a 50 mL beaker, adjusting the water content with deionized water to maintain the amount of water at approximately 65%, and weighing the beaker as a constant value at this time. Lye and the soil-filled beaker were placed in an incubation bottle to be sealed and cultured in the dark. The lye was a solution of 0.1 mol L^−1^ NaOH. The lye was removed and titrated with dilute acid at 1, 3, 5, 7, 9, 16, 23, 30, 37, 44, 51, and 58 days of incubation respectively. The dilute acid was a solution of 0.1 mol L^−1^ HCl. A volume of two mL of one mol L^−1^ BaCl_2_ solution and 2 drops of phenolphthalein indicator were added to the lye before each titration. After the dilute acid titration had been completed, a new lye solution was replaced. At this time, the beaker that contained the soil was removed and weighed, and deionized water was added to keep it at a constant weight before incubation.

### Data analysis

The rate of soil organic carbon (SOC) mineralization was calculated as described by Wang et al. (2014). After the indoor incubation was completed, the first-order kinetic equation in Origin 2017 software (OriginLab, Northampton, MA, USA) was used to fit the SOC cumulative mineralization parameters to obtain the potential mineralizable organic carbon (C_0_) and turnover rate constant (k) (Stanford & Smith, 1972). The formula for first-order kinetic model is shown as follows:

(1) \begin{eqnarray*}{\mathrm{C}}_{\mathrm{t}}={\mathrm{C}}_{0}(1-{\mathrm{e}}^{\mathrm{- kt}})\end{eqnarray*}

where C_t_ is the cumulative mineralization of SOC (mg kg^−1^); C_0_ is the potential mineralizable organic carbon (mg kg^−1^), and k is the turnover rate constant (d^−1^). The semi-turnover period was T_1∕2_ = ln2/k (d).

The soil depth and compound ratio were the two factors in this study. The data from soil physical and chemical properties and mineralization were subjected to a Duncan multiple comparison and analysis of variance (ANOVA) using Microsoft Excel 2010 (Redmond, WA, USA) and SPSS 20.0 (IBM, Inc., Armonk, NY, USA).

## Results

### Soil physical and chemical properties

The combined effect of the two factors of soil depth and compound ratio had a significant impact on the SOC, AK, sand, clay and texture (Table 1). The SOC, TN and clay contents of the S1, S2, and S3 treatments were significantly higher in the 0–10 cm soil depth than those of the CK treatment (*P* < 0.05), and there was no significant difference in the content of AP between treatments (*P* > 0.05). The contents of AK and silt in the S2 and S3 treatments were significantly higher than those in the CK treatment, and the content of sand was lower in the S2 and S3 treatments. There was no significant difference between the contents of SOC and AP in the 10–20 cm soil depth treatment, and the contents of TN and AK of the S3 treatment were significantly higher than those of the CK. The content of sand decreased significantly with the increase in proportion of soft rock, while the contents of silt and clay increased significantly in in the 10–20 cm soil depth treatment. During the 20–30 cm soil depth, there was no significant difference in the contents of SOC, TN, AP and AK among the treatments. The sand content of S2 treatment decreased significantly, and the clay content increased significantly. All of the nutrients were found at higher concentrations in the 0–10 cm soil depth. With the change in composition of soil particles in the compound soil, the trend of change of soil texture was sand-loamy sand-sandy loam. It was apparent that the content of soil nutrients increased significantly under the S2 and S3 treatments, with a higher content in the 0–10 cm soil depth. The soil texture developed benign.

**Table 1:** Soil nutrient index and particle composition.

Soil depth (cm)	Compound ratio	SOC (g kg^−1^)	TN (g kg^−1^)	AP (mg kg^−1^)	AK (mg kg^−1^)	Sand (%)	Silt (%)	Clay (%)	Texture
0–10	CK	2.95 ± 0.14 bA	0.20 ± 0.02 bA	18.4 ± 0.40 aA	50.00 ± 5.00 cA	100 ± 0 aA	0 ± 0 cA	0 ± 0 bB	Sand
	S1	5.57 ± 1.71 aA	0.48 ± 0.14 aA	20.43 ± 2.74 aA	65.00 ± 10.66 bcA	84.42 ± 4.20 bA	14.80 ± 2.91 bA	0.78 ± 0.29 a A	Loamy sand
	S2	4.54 ± 0.81 aA	0.48 ± 0.04 aA	17.33 ± 2.98 aA	111.67 ± 4.62 aA	79.83 ± 5.35 bcA	19.13 ± 4.01 abA	1.04 ± 0.34 aA	Loamy sand
	S3	5.13 ± 0.74 aA	0.55 ± 0.08 aA	16.57 ± 3.01 aA	100.00 ± 6.00 abA	72.32 ± 5.20 cB	26.14 ± 4.83 aA	1.54 ± 0.38 aA	Loamy sand
10–20	CK	3.98 ± 0.06 aA	0.34 ± 0.06 bA	11.93 ± 1.05 aA	44.00 ± 5.00 bA	90.95 ± 0.05 aB	8.93 ± 0.20 dA	0.12 ± 0.03 dB	Sand
	S1	4.38 ± 012 aA	0.38 ± 0.12 abAB	14.77 ± 0.68 aAB	58.00 ± 12.49 abA	84.72 ± 1.44 bA	14.41 ± 1.32 cA	0.87 ± 0.13 cA	Loamy sand
	S2	4.42 ± 0.17 aA	0.38 ± 0.11 abA	13.67 ± 2.99 aA	60.33 ± 5.51 abB	75.87 ± 0.76 cA	22.66 ± 0.81 bA	1.47 ± 0.07 bA	Loamy sand
	S3	4.59 ± 0.82 aA	0.54 ± 0.10 aA	13.76 ± 2.62 aA	69.00 ± 5.57 aB	68.63 ± 3.66 dB	29.33 ± 3.37 aA	2.04 ± 0.30 aA	Sandy loam
20–30	CK	4.01 ± 0.02 aA	0.26 ± 0.04 aA	9.10 ± 0.60 aA	50.01 ± 3.25 aA	87.34 ± 0.65 aB	11.96 ± 1.05 abA	0.70 ± 0.20 cA	Sand
	S1	2.50 ± 0.96 aB	0.24 ± 0.06 aB	6.80 ± 0.61 aB	41.33 ± 8.50 aA	86.53 ± 5.11 aB	12.42 ± 0.92 abA	1.05 ± 0.28 abB	Sand
	S2	2.87 ± 0.21 aA	0.32 ± 0.07 aA	7.50 ± 2.09 aA	38.67 ± 4.04 aB	81.53 ± 4.15 bA	15.01 ± 3.81 aA	1.46 ± 0.30 aA	Loamy sand
	S3	2.58 ± 0.58 aB	0.30 ± 0.15 aB	7.57 ± 1.35 aA	40.00 ± 5.58 aC	83.42 ± 6.20 abA	15.57 ± 2.84 aA	1.01 ± 0.31 bcB	Loamy sand
Soil depth × Compound ratio		*	/	/	**	**	/	**	**

**Notes.**

SOC refers to soil organic carbon TN refers to soil total nitrogen AP refers to soil available phosphorus AK refers to soil available potassium

Mean ±  standard deviation, lowercase letters indicate significant differences between different treatments under the same soil depth, capital letters indicate significant differences between different soil depth under the same treatment (*P* < 0.05).

### Rate of SOC mineralization

**Figure 2:**
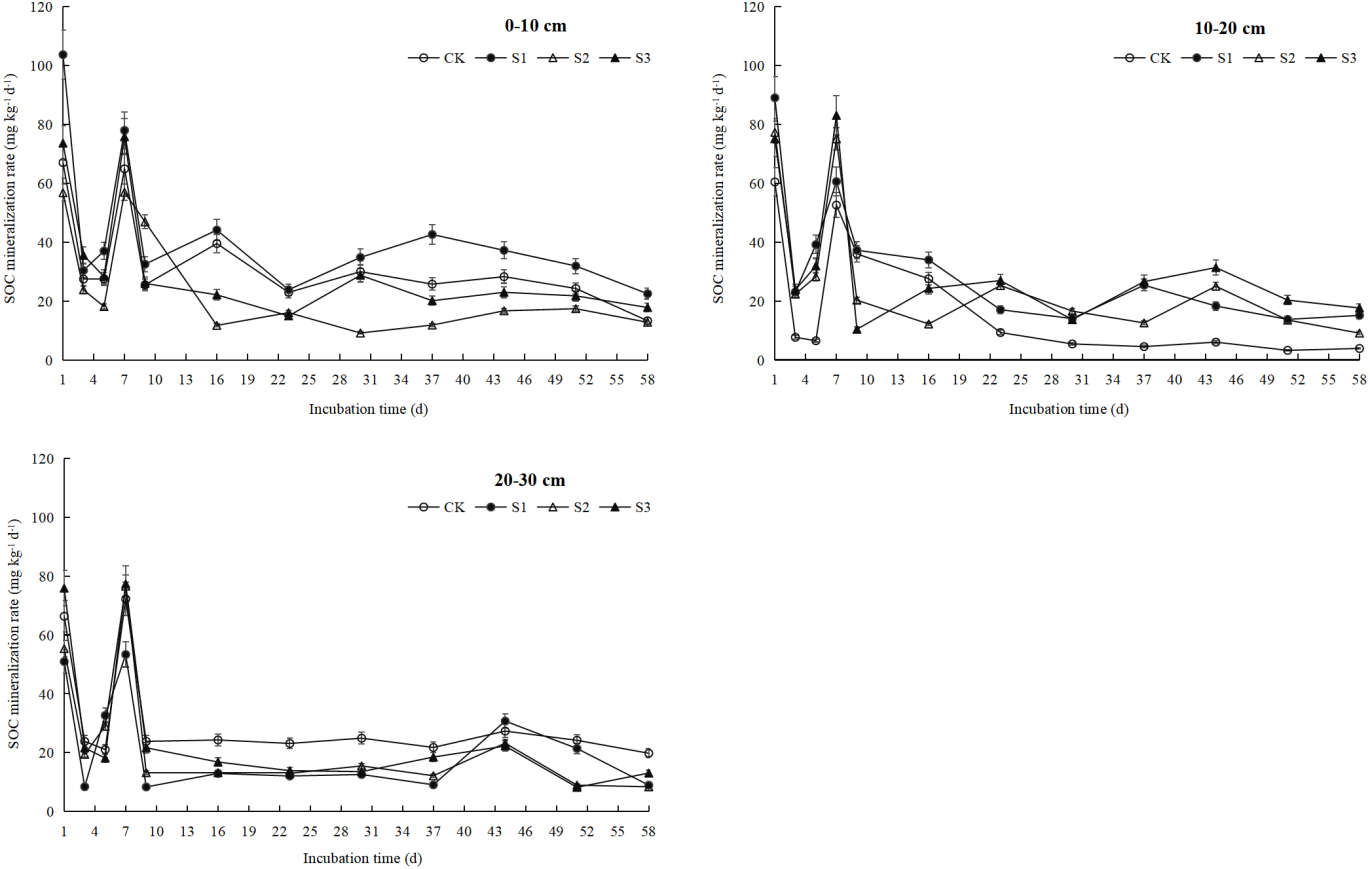
SOC mineralization rate under different soil layers. CK represents the volume ratio of soft rock to sand is 0:1; S1 represents a volume ratio of soft rock to sand of 1:5; S2 represents a volume ratio of soft rock to sand of 1:2; S3 represents the volumetric ratio of soft rock to sand is 1:1.

The rate of mineralization of different types of organic carbon in the compound soils changed with the incubation time, revealing clear differences in their characteristics depending on the stage. During the early stage of incubation, the rate of SOC mineralization was unstable, and it first decreased and then increased (Fig. 2). The rate of mineralization of organic carbon of each soil depth reached its peak on day 7, and the range of variation was 56.99–78.05 mg kg^−1^ d^−1^ (0–10 cm), 52.64–83.13 mg kg^−1^ d^−1^ (10–20 cm), and 53.36–77.32 mg kg^−1^ d^−1^ (20–30 cm). The degree of variation could be owing to the high content of easily mineralized organic carbon in the soil, along with the decomposition of light organic carbon; a large amount of nutrients required for microbial metabolism are produced, which leads to an increase in the activity of soil microorganisms and rate of SOC mineralization (Liu & Song, 2008; Zhang et al., 2021). The rate of mineralization of SOC slowly declined and gradually stabilized during the latter period of experiment. At the end of culture, the rates of SOC mineralization of S1, S2, and S3 treatments in the 0–10 cm soil depth were 22.51 mg kg^−1^ d^−1^, 12.80 mg kg^−1^ d^−1^, and 17.83 mg kg^−1^ d^−1^, respectively. The rates of mineralization of the CK and S2 were basically the same, while those of S1 and S3 increased by 12.71% and 33.96%, respectively, compared with those of the CK. The average values of rate of SOC mineralization in the 10–20 cm and 20–30 cm soil depths were basically the same, but both were less than that in the 0–10 cm soil depth. In the 10–20 cm soil depth, the rate of SOC mineralization of S1, S2, and S3 treatments increased by 288.36%, 133.33%, and 351.72%, respectively, compared with that of the CK. At the 20–30 cm soil depth, the rates of mineralization of SOC of the S1, S2, and S3 treatments were lower than that of the CK treatment.

### SOC cumulative mineralization (C_t_)

**Figure 3:**
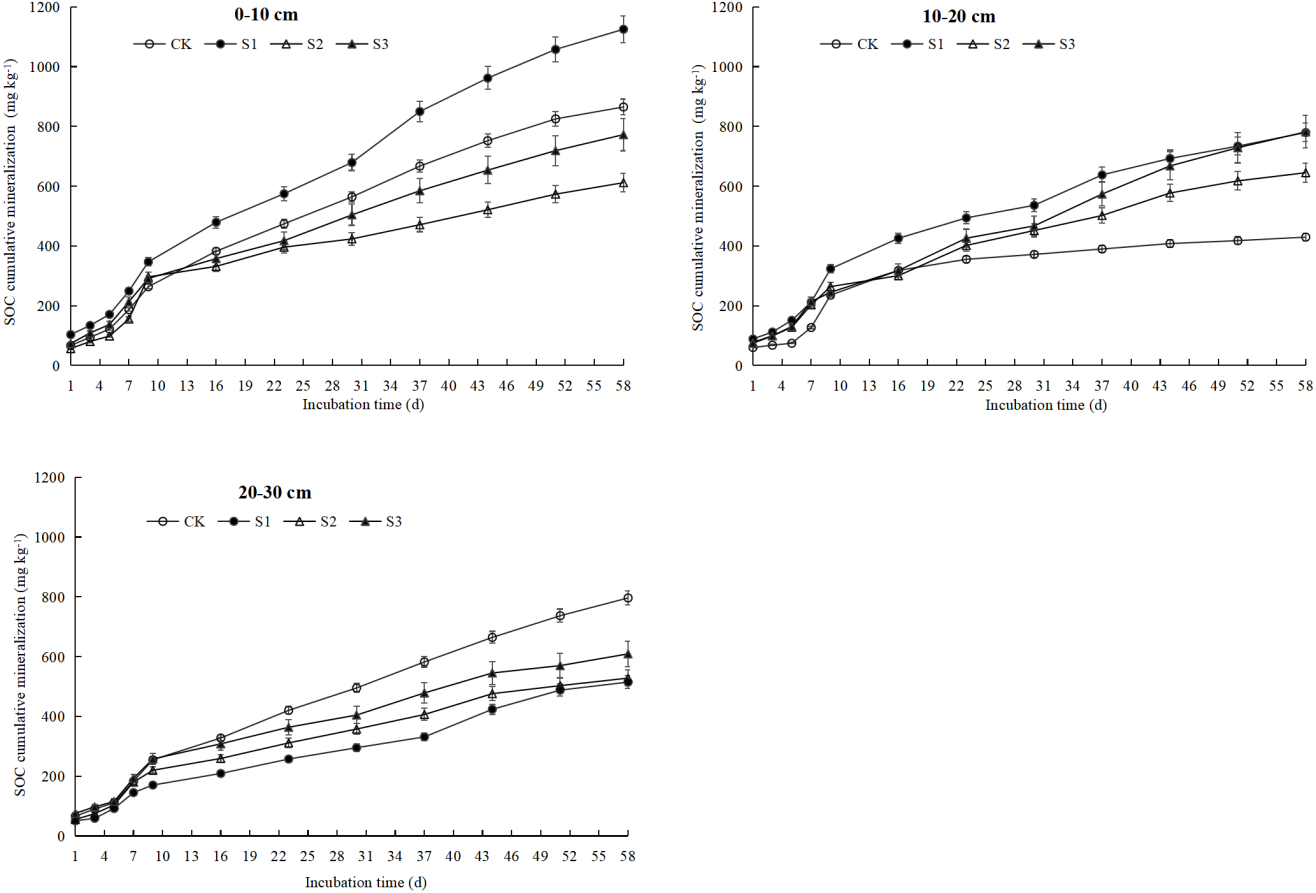
SOC cumulative mineralization of each treatment under different soil layers. CK represents the volume ratio of soft rock to sand is 0:1; S1 represents a volume ratio of soft rock to sand of 1:5; S2 represents a volume ratio of soft rock to sand of 1:2; S3 represents the volumetric ratio of soft rock to sand is 1:1.

The C_t_ in each soil depth increased exponentially with the incubation time, and the average value gradually decreased as the soil depth deepened. The specific performance was 0–10 cm (843.72 mg kg^−1^) > 10–20 cm (659.16 mg kg^−1^) > 20–30 cm (612.30 mg kg^−1^) (Fig. 3). In the same soil depth, the C_t_ differed between treatments with different compound ratios. In the 0–10 cm soil depth, the S1 treatment had the largest C_t_, which was 1,125.17 mg kg^−1^, followed by the CK, S3, and S2 treatments. In the 10–20 cm soil depth, the S3 treatment had the largest C_t_, which was 781.91 mg kg^−1^, followed by those of the S1, S2, and CK treatments. In the 20–30 cm soil depth, the CK treatment had the largest C_t_, which was 796.49 mg kg^−1^, followed by the S3, S2, and S1 treatments. When all the soil depths were factored into account, the average cumulative mineralization of organic carbon was the largest in S1 treatment, which was 806.62 mg kg^−1^, followed by the S3, CK and S2 treatments, and the cumulative mineralization of organic carbon was 721.30 mg kg^−1^, 697.14 mg kg^−1^, and 595.19 mg kg^−1^, respectively.

### Cumulative rate of SOC mineralization

**Figure 4:**
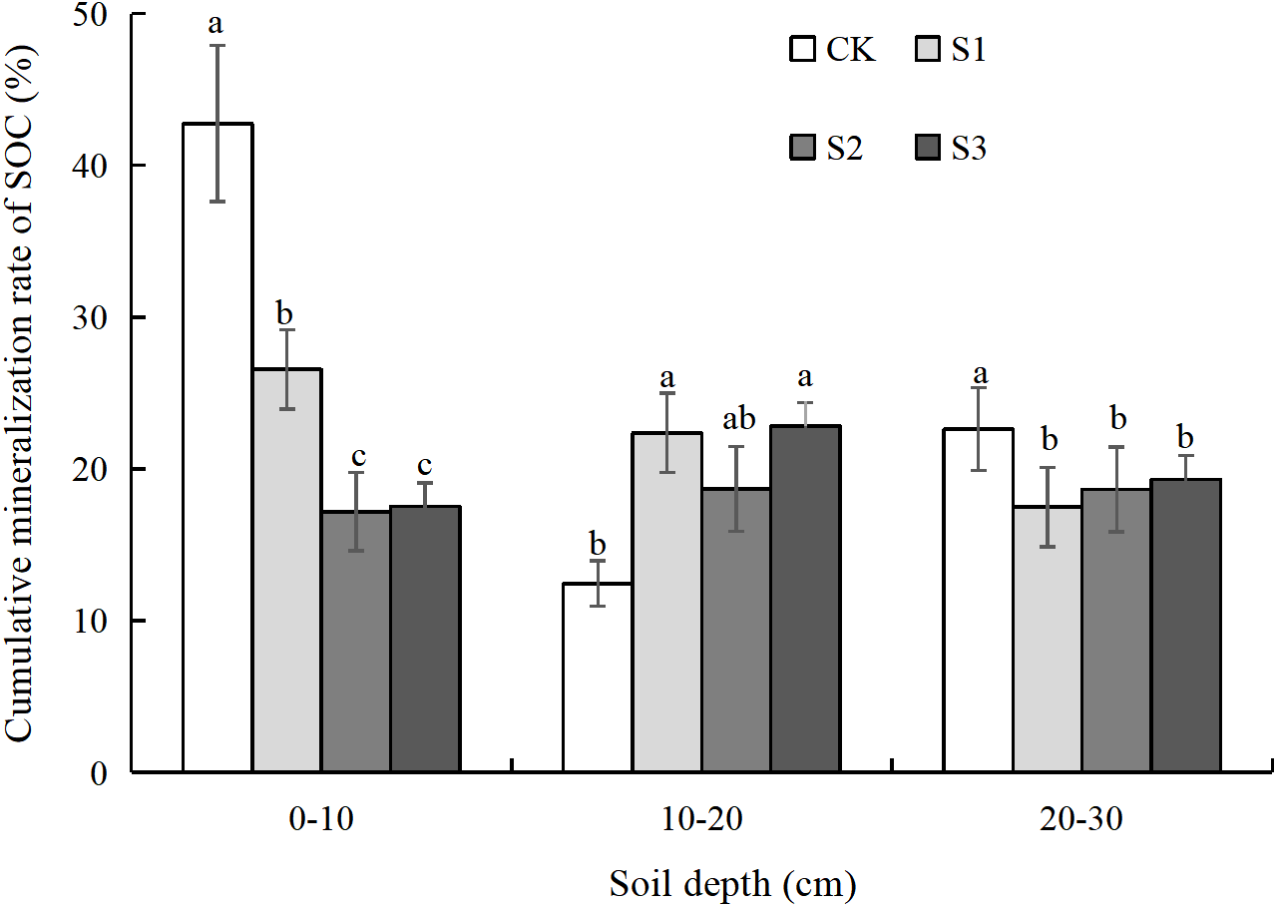
Cumulative mineralization rate of SOC under different soil layers. CK represents the volume ratio of soft rock to sand is 0:1; S1 represents a volume ratio of soft rock to sand of 1:5; S2 represents a volume ratio of soft rock to sand of 1:2; S3 represents the volumetric ratio of soft rock to sand is 1:1. Lowercase letters indicate significant differences at the 5% level between different treatments in the same soil layer.

The accumulated rate of mineralization (C_r_) of the SOC in compound soils of different soil depths differed to some extent. The C_r_ in the 0–10 cm (26.01%) soil depth was significantly higher than that at the 10–20 cm (19.08%) and 20–30 cm (19.52%) soil depths (Fig. 4). In the 0–10 cm soil depth, the ratios of S1, S2, and S3 were reduced by 1.61-, 2.49-, and 2.44-fold compared with the CK, respectively. Compared with the S1 treatment, the C_r_ of SOC under S2 and S3 treatments was significantly reduced by 1.55- and 1.52-fold. In the 10–20 cm soil depth, the C_r_ of SOC treated by S1, S2 and S3 did not differ significantly but increased to some extent compared with that treated by the CK. In the 20–30 cm soil depth, there was no significant difference in the C_r_ of SOC treated by S1, S2, and S3, but it was significantly reduced by 1.17−1.29-fold compared with that treated by the CK. It was apparent that the S2 and S3 treatments had a lower C_r_, which is beneficial to carbon storage, and the effect at the 10–20 cm soil depth was even better.

### SOC mineralization kinetic equation fitting

Based on the CO_2_-C release measured at each stage of incubation in the 58 d constant room temperature, the C_t_ in soil depth with different compound ratios of 0–10 cm, 10–20 cm and 20–30 cm was fitted using a first-order kinetic equation (C_t_ = C_0_[1–e-^kt^]). The results showed that the fitting effect was excellent (*R*^2^ > 0.96), and fitting parameters, such as C_0_, k and T_1∕2_, were obtained (Table 2). Among all the soil depths, the 0–10 cm soil depth has the largest C_0_. The C_0_ and C_t_ of 0–10 cm and 10–20 cm soil depths have the same tendency to change, and the C_0_ value of the 20–30 cm soil depth was the largest when treated with S1 and S3. The k value was the largest in the 10–20 cm soil depth, and there was no significant difference between the 0–10 cm and 20–30 cm soil depths. The k value of the S1 treatment in the 0–10 cm and 20–30 cm soil depths was significantly lower than those of the S2 and S3 treatments. The tendency of T_1∕2_ to change was opposite to the value of k, and the T_1∕2_ of 0–10 cm and 20–30 cm soil depths were larger. The C_0_/SOC in the 10–20 cm soil depth was the smallest (25.47%), and the C_0_/SOC in the S2 and S3 treatments was small (21.48% and 28.27%, respectively). In summary, the mineralization of the 10–20 cm soil depth was relatively small, and the ability of the S2 and S3 treatments to sequester carbon was relatively strong.

**Table 2:** Parameters of the first-order kinetics for the SOC mineralization.

Soil depth (cm)	Compound ratio	C_0_ (mg kg^−1^)	k (×10^−3^ d^−1^)	T_1∕2_(d)	C_0_/SOC (%)	R^2^
0–10	CK	1561.71 ± 136.72 bA	27.10 ± 0.33 cB	25.5 ± 2.13 aA	77.19	0.9971
	S1	2242.29 ± 207.55 aA	23.05 ± 0.55 cB	30.10 ± 1.26 aA	52.93	0.9911
	S2	684.04 ± 46.13 dAB	63.52 ± 0.85 aA	10.92 ± 0.08 bcA	19.21	0.9835
	S3	1048.51 ± 119.79 cB	41.46 ± 0.76 bB	16.75 ± 0.09 bB	23.78	0.9840
10–20	CK	454.49 ± 18.64 dB	99.28 ± 0.11 aA	6.98 ± 0.21 bcB	13.16	0.9813
	S1	912.92 ± 46.96 bB	59.07 ± 0.82 bA	11.73 ± 0.10 bB	26.20	0.9917
	S2	785.99 ± 65.68 bcA	53.84 ± 0.58 bB	12.87 ± 0.63 bA	22.76	0.9824
	S3	1362.71 ± 73.93 aA	27.57 ± 0.876 cC	25.14 ± 0.73 aA	39.79	0.9826
20–30	CK	1499.94 ± 214.65 aA	24.56 ± 0.47 bB	28.22 ± 0.15 aA	42.61	0.9931
	S1	1008.93 ± 181.87 bB	22.68 ± 1.21 bB	30.56 ± 0.61 aA	34.28	0.9610
	S2	637.18 ± 62.45 cB	54.30 ± 0.97 aB	12.77 ± 0.71 bA	22.48	0.9754
	S3	717.43 ± 62.57 cC	56.72 ± 0.93 aA	12.22 ± 0.75 bBC	22.74	0.9778

**Notes.**

Mean ±  standard deviation, lowercase letters indicate significant differences between different treatments under the same soil layer, capital letters indicate significant differences between different soil depth under the same treatment (*P* < 0.05).

### Characteristics of the variation of DOC and SMBC during SOC mineralization

Before and after the incubation, the average DOC content decreased with the deepening of the soil depth and increased with the increase in the proportion of soft rock. The average content of SMBC was the largest in the 10–20 cm soil depth before and after incubation, and the increase paralleled that of the proportion of soft rock. With the extension of the incubation time, the contents of DOC and SMBC tended to decrease compared with the initial content. After the incubation, the contents of DOC in the 0–10 cm, 10–20 cm, and 20–30 cm soil depth decreased by 17.76–23.21%, 19.86–24.46%, and 14.99–20.58%, respectively, compared with those before incubation. The contents of SMBC were reduced by 14.55–21.54%, 12.69–20.01% and 19.34–20.99%, respectively, compared with before incubation (Table 3). In the 0–10 cm soil depth, there was no significant difference in the content of DOC between the treatments before and after the incubation, and the content of SMBC was the largest in the S3 treatment. In the 10–20 cm soil depth, the contents of DOC of the S1, S2, and S3 treatments before and after the incubation were significantly higher than that of the CK treatment, and the content of SMBC was the highest in S2 treatment. In the 20–30 cm soil depth, the changes of DOC and SMBC before and after the incubation were the same, and the contents of both S2 and S3 treatments were higher. It is apparent that the contents of DOC and SMBC of the S2 and S3 treatments have a greater tendency to change, and the mineralization was stronger, particularly in the 10–20 cm soil depth.

**Table 3:** The initial content of DOC and SMBC in different compound ratios, depth and its changes after incubation.

Soil depth (cm)	Compound ratio	DOC (mg kg^−1^)				SMBC (mg kg^−1^)		
		Before incubation	After incubation	Decrease value		Before incubation	After incubation	Decrease value
0–10	CK	5.01 ± 0.93 aA	4.12 ± 0.53 aA	0.89 ± 0.06 bA		50.32 ± 2.41 dB	39.48 ± 2.39 dB	10.84 ± 1.05 bA
	S1	5.60 ± 1.61 aA	4.30 ± 0.75 aA	1.30 ± 0.32 aA		60.12 ± 1.55 cB	48.32 ± 5.36 cB	11.80 ± 0.96 abB
	S2	5.84 ± 1.13 aA	4.57 ± 0.31 aA	1.27 ± 0.13 aA		76.84 ± 1.58 bB	65.66 ± 1.86 bB	11.18 ± 2.12 abC
	S3	5.82 ± 1.32 aA	4.68 ± 1.06 aA	1.15 ± 0.08 aA		95.34 ± 1.04 aA	81.12 ± 3.75 aA	14.22 ± 1.22 aB
10–20	CK	3.53 ± 0.82 bB	2.79 ± 0.75 bB	0.74 ± 0.04 bA		55.22 ± 2.42 cAB	44.72 ± 2.89 cAB	10.50 ± 1.45 cA
	S1	5.56 ± 0.44 aA	4.20 ± 1.52 aA	1.36 ± 0.03 aA		80.38 ± 1.60 bA	64.30 ± 4.06 bA	16.08 ± 1.37 bA
	S2	5.19 ± 0.15 aA	4.07 ± 1.31 aA	1.12 ± 0.09 aA		95.50 ± 1.46 aA	76.40 ± 2.01 aA	19.10 ± 1.03 aA
	S3	5.89 ± 0.36 aA	4.72 ± 0.47 aA	1.17 ± 0.21 aA		85.92 ± 1.04 bB	75.02 ± 2.37 aB	10.90 ± 1.28 cC
20–30	CK	3.50 ± 0.62 bB	2.78 ± 0.29 bB	0.72 ± 0.22 abA		60.66 ± 2.42 bA	48.58 ± 4.34 bA	12.08 ± 2.14 bA
	S1	3.87 ± 0.96 bB	3.29 ± 0.62 bB	0.58 ± 0.07 bB		60.80 ± 1.40 bB	49.04 ± 3.32 bB	11.76 ± 2.81 bB
	S2	5.07 ± 1.51 aA	4.16 ± 1.14 aA	0.91 ± 0.13 aAB		80.96 ± 1.38 aB	64.76 ± 2.01 aB	16.20 ± 1.73 aB
	S3	5.27 ± 1.31 aA	4.39 ± 0.86 aA	0.87 ± 0.05 aAB		81.92 ± 1.04 aB	64.72 ± 3.23 aC	17.20 ± 1.97 aA

**Notes.**

Mean ±  standard deviation, lowercase letters indicate significant differences between different treatments under the same soil depth, capital letters indicate significant differences between different soil depth under the same treatment (*P* < 0.05).

### Relationship between the parameters of SOC mineralization and physicochemical properties

Through the correlation analysis of the C_0_ and k values of three soil depths and four soils with different compound ratios and soil physicochemical properties, the results showed that there was no significant correlation between the C_0_ and k values at the 0–10 cm soil depth and the soil physicochemical properties (Table 4). As the soil depth deepened, the correlation became clearer. At the 10–20 cm soil depth, the C_0_ value had no significant correlation with the soil physicochemical properties, but the k value had a significant negative correlation with the contents of SOC and silt and an extremely significant negative correlation with the AK content. In the 20–30 cm soil depth, the C_0_ value significantly positively correlated with the AK content. The k value significantly positively correlated with the contents of TN and DOC and was extremely significantly positively correlated with the content of SMBC. It showed that the influence of soil depth and compound ratio on C_0_ and K of the 0–10 cm soil depth was not significant and had little effect on mineralization, while the influence of 10–20 cm and 20–30 cm soil depths on the mineralization of organic carbon was higher.

**Table 4:** Correlation analysis of soil C_0_, k and soil physicochemical properties.

Soil depth	Items	SOC	TN	AP	AK	Sand	Silt	Clay	DOC	SMBC
0–10 cm	C_0_	0.1737	−0.2162	0.9132	−0.8016	0.4236	−0.4225	−0.4404	−0.4414	−0.6304
	k	0.0623	0.3904	−0.6876	0.8958	−0.5115	0.5120	0.5013	0.6317	0.5740
10–20 cm	C_0_	0.9189	0.9412	0.6180	0.9450	−0.8682	0.8655	0.8917	0.8932	0.6163
	k	−0.9925^*^	−0.8484	−0.6912	−0.9997^**^	0.9406	−0.9372^*^	−0.9704	−0.9477	−0.8252
20–30 cm	C_0_	0.8130	−0.7206	0.7116	0.9742^*^	−0.1229	−0.517	−0.5488	−0.9181	−0.8531
	k	−0.4104	0.9552^*^	−0.2057	−0.6785	0.5830	0.8442	0.8487	0.9761^*^	0.9987^**^

**Notes.**

* Significance level at < 0.05.

** Significance level at < 0.01.

## Discussion

With the extension of incubation time, the C_t_ content increased continuously. The process of mineralization of SOC of different compound ratios can be roughly divided into three stages: stress change stage (1–7 d), rapid mineralization stage (7–9 d) and slow mineralization stage (9–58 d), which was similar to the results of previous research (Marinari et al., 2010). In this study, the mineralization rate was higher on the first day of incubation, primarily because the water added to the soil at the initial stage of incubation stimulated the soil microorganisms (Tang et al., 2017). Studies have confirmed the following: water stimulates soil microorganisms, which will affect the soil microbial community structure and microbial activity, and then affect the dynamic process of SOC mineralization (Wang et al., 2011). In the latter period of incubation, as the content of easily mineralizable carbon decreases, the rate of SOC mineralization decreases, and the microorganisms begin to decompose relatively stable organic carbon components (Guo, Han & Li, 2020). Therefore, the SOC mineralization rate gradually decreases and then stabilizes.

Different soil physical and chemical properties have mineralization levels that vary. This study found that the C_t_ value of S1 treatment was the largest, followed by S3, and the S2 treatment was the smallest. This difference was owing to the higher proportion of soil nutrient content and active organic carbon the S1 treatment. The rate of SOC mineralization was consistent with the change in C_t_. Zha et al. (2014) pointed out that SOC was the main driving factor of soil fertility and has a significant impact on the mineralization process. The results of this study also showed that there was a significant negative correlation between SOC and the k value (Table 4), indicating that the content of SOC was one of the main factors that affected the difference in SOC mineralization (Zhang et al., 2016). Among the many factors that affected the intensity of SOC mineralization, the contents of TN and AK also had a substantial influence on the mineralization of SOC, and it had a significant correlation with the C_0_ and k values. This was consistent with the conclusion that the organic carbon mineralization studied by Li et al. (2011) had a significant positive correlation with the initial content of TN. Both C_r_ andC_0_/SOC were the ratio of soil easily decomposable organic carbon to total organic carbon, which can visually demonstrate the relative stability of SOC (Yang et al., 2019). The results of this study indicated that the C_r_ andC_0_/SOC values treated by CK and S1 were relatively large, with a strong ability for mineralization and minimal storage of organic carbon. Under the premise of maintaining a certain SOC content, the higher ability to mineralize SOC indicated that the organic carbon was more unstable, which was not conducive to SOC fixation (Qiu et al., 2021). The intensity of SOC mineralization generally decreased with the deepening of the soil depth, i.e., the ability of the 0–10 cm soil depth to mineralize SOC was high, followed by 10–20 cm and 20–30 cm. The lower C_r_ and C_0_/SOC values in the 10–20 cm soil depth indicated that the active organic carbon in the surface layer was higher, and the SOC in the bottom layer was more stable. This could be because the 10–30 cm bottom soil was more stable than the 0–10 cm surface soil, and the short-term changes were small. In addition to the effects of mineralization of organic carbon, there are many other factors that affect the ability of soil to sequester carbon, including land use patterns, vegetation types and human disturbances (Ghimire & Khanal, 2020). Therefore, the factors that influence the sequestration of soil carbon urgently merit further study.

With the increase in soil depth, the DOC and SMBC exhibited a trend that was closely related to the k value. Since the DOC can be more easily decomposed and utilized by microorganisms, organic carbon must enter the soil solution to be converted into DOC before it is converted to CO_2_, and the soil moisture moves downward along with it Tang et al. (2017). Therefore, the 20–30 cm soil depth showed a correlation between DOC and SMBC with the mineralization of SOC. Li, Zhang & Chen (2004) also found that the DOC was closely related to the mineralization of SOC and proposed that increasing the content of soil water can increase the content of DOC in the soil and increase the substrates available to soil microorganisms, thereby promoting the mineralization of SOC. Therefore, the dynamics and turnover of DOC content were closely related to the mineralization of SOC. SMBC is an important carbon source that composes soil humus, and it is substantially significant to the turnover of SOC and the ecological environment (Guo, Han & Li, 2020). With the extension of the incubation time, the contents of SMBC and DOC in different treatments and soil depth decreased to varying degrees. This phenomenon indicates that during the process of cultivation, soil active organic carbon is used as a carbon source for SOC mineralization, which is decomposed and utilized by microorganisms and releases CO_2_ during respiration, resulting in a decrease in the active organic carbon content in the soil, which directly affects the cumulative release of CO_2_. In summary, the mineralization of SOC was affected by soil depth, compound ratio and active organic carbon, and changes under the combined action of these factors. In the future, it will be necessary to continue to strengthen the research on functional microorganisms and reveal the microbial mechanism of organic carbon mineralization.

## Conclusions

The trend of change of soil organic carbon (SOC) mineralization rate curve and cumulative mineralization curve at different soil depths of soft rock and sand compound soil was basically the same, and both showed the characteristics of first decreasing, then increasing and finally stabilizing at a certain level. However, with the deepening of soil depth, the rate of mineralization of SOC and the cumulative mineralization of compound soil gradually decreased. These characteristics may be affected by soil properties, such as organic carbon vertical input, soil active organic carbon composition, and the contents of total nitrogen (TN) and available potassium (AK). In concert with the process of SOC mineralization, the soil microbial biomass carbon and dissolved organic carbon content of different treatments decreased compared with the initial content, and the amplitude of change was larger in the 20–30 cm and 10–20 cm soil depths. The surface soil stability was poor, and the rate of mineralization of SOC and C_t_ decreased with the increase in soil depth, and the C_r_ and C_0_/SOC values in the 10–20 cm soil depth were smaller. Compared with the other treatments, those of S2 and S3 can reduce the loss of carbon. Deep SOC has a slow rate of mineralization and a small amount of accumulated mineralization. This process was affected by the contents of TN and AK, which may become a carbon sink or a carbon source. Therefore, when conducting research on the global carbon cycle, deep SOC and surface SOC should be placed in an equally important position.

##  Supplemental Information

10.7717/peerj.11572/supp-1 Supplemental Information 1 Raw data Soil physicochemical properties and organic carbon mineralization rate, cumulative mineralization amount, cumulative mineralization rate and kinetic equation fitting parameters. The organic carbon mineralization raw data were obtained under the conditions of 58 days of indoor incubation. CK represents the volume ratio of soft rock to sand is 0:1; S1 represents a volume ratio of soft rock to sand of 1:5; S2 represents a volume ratio of soft rock to sand of 1:2; S3 represents the volumetric ratio of soft rock to sand is 1:1.

## Additional Information and Declarations

### Data Availability

The following information was supplied regarding data availability: The raw data are available in the Supplemental File.
